# Evaluation of the Food Barrier and Mechanical Properties of Carrageenan‐Starch Composite Films

**DOI:** 10.1002/fsn3.4664

**Published:** 2024-12-09

**Authors:** E. S. Madivoli, J. Kisato, P. K. Kimani, K Kamau

**Affiliations:** ^1^ Department of Chemistry Jomo Kenyatta University of Agriculture and Technology Nairobi Kenya; ^2^ Department of Physics and Biophysics University of Warmia and Mazury in Olsztyn Olsztyn Poland; ^3^ Department of Fashion and Design Kenyatta University Nairobi Kenya; ^4^ Department of Engineering Science, Graduate School of Engineering Gifu University Gifu Japan

**Keywords:** edible blends, food packaging, seaweed, starch

## Abstract

Single use plastics are a leading source of microplastics that have been detected along the food chain. This study evaluated the potential of starch (ST) and carrageenan (CRG) in packaging film formulation. CRG isolated from the seaweed (SW) *Eucheuma denticulatam* was blended with starch and cast to obtain films whose moisture content (MC), total soluble matter (TSM), degree of solubility (DS), water vapor permeability (WVP), opacity (O), contact angles (CA), moisture absorption (MA), and percent elongation (PE) were evaluated. The films’ morphology, crystallinity, opacity, thermal profile, and functional groups were then studied by scanning electron microscopy, powder diffraction, UV–Vis, thermal gravimetry, and infrared spectroscopy. From the results obtained, the SWF films exhibited a higher MC, DS, and TSM than CRG and CRG‐ST films but lower DC values. The PE of CRG films was lower than that of SWF (30%) though incorporation of ST increased the PE of CRG‐ST. However, SWF films had WVP of 2.25 × 10^−7^ gs^−1^m^−1^Pa^−1^, compared to 3.65 × 10^−7^ gs^−1^m^−1^Pa^−1^ of CRG, 2.73 × 10^−7^ gs^−1^m^−1^Pa^−1^ of CRG‐ST and a moisture absorption of 29.29 ± 3.5 as compared to 17.29 ± 0.87 of CRG and 23.80% ± 4.12% of CRG‐ST. The opacities were found to be 41.02, 79.89, and 42.23 for SWF, CRG, CRG‐ST while the contact angles were found to be 72.86, 80.93, 65.57 for SWF, CRG, and CRG‐ST, respectively. Moreover, the films were impermeable to vegetable oil, had carbohydrate functional groups, good thermal stabilities, and trace micronutrients. In conclusion, this study formulated packaging films with enhanced food barrier and mechanical properties that can potentially replace single use packaging films.

## Graphical abstract

Carrageenan based composite films for packing materials toward sustainable economies.

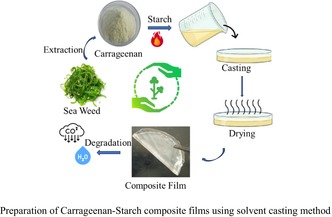

## 1 Introduction

There is an increasing need to find biodegradable alternatives to single use plastics, which are a major contributor to microplastics currently being detected everywhere. These microplastics have been found in the ocean, sea food, breast milk, drinking water, in the atmosphere as wafting particles, in rain drops, and in human blood, placenta, and arteries (Danopoulos, Twiddy, and Rotchell 2020; Eriksen et al. 2014; Kumar et al. 2022; Leslie et al. 2022; Yang, Chen, and Wang 2021). Until recently, the impact of microplastics pollution on human health was not fully understood up until data that linked the presence of microplastics in human arteries to increased risk of heart attack, stroke, and even death was published (Marfella et al. 2024). Moreover, a recent study reported that of the 16,000 chemicals used in various plastics as raw ingredients and additives such as stabilizers and colorants, at least 4000 of these are persistent, bio accumulative, mobile, and/or toxic (Wagner et al. 2024). Although there is a lot about the environmental impact and health hazards posed by microplastics that we do not yet understand, the available little information is alarming (Dmitrenko et al. 2023; Nieto‐Suaza et al. 2019; Sedayu, Cran, and Bigger 2018; Wan Yahaya et al. 2023). Moreover, current patterns of production, use, and disposal of petroleum based single use plastics are not sustainable (Leslie et al. 2022; Okunola et al. 2019; Worm et al. 2017). In response to the growing problem of plastic pollution and production, it is now increasingly paramount that more biodegradable and sustainable alternatives be developed from renewable resources such as starch, cellulose, carrageenan, and other biopolymers in line with the principles of a circular bioeconomy. More specifically, there is need to explore the applicability of these biopolymer as alternative starting material for food packaging materials to replace single use plastics (Okunola et al. 2019). To achieve this, their biodegradability, recyclability, antimicrobial, and mechanical properties need to be understood (Castro‐García et al. 2022; Madivoli 2023). For food safety concerns, evidence suggests that incorporation of antimicrobial moieties within the bioplastics matrices prevents food contamination through microbial inhibition (Shankar, Wang, and Rhim 2016; Wan Yahaya et al. 2023; Wu et al. 2019). On the other hand, it has been reported that blending two or more biopolymers improves the mechanical properties of the resultant composite through a synergistic effect realized when two or more biopolymers with different characteristics such as cellulose, chitosan and starch are used interchangeably as nanofillers (Mohanty, Misra, and Drzal 2002; Rhim, Park, and Ha 2013; Visakh, Mathew, and Thomas 2013). This synergistic effect not only improves the mechanical properties of the resultant food packaging materials, but it also improves their food barrier properties, water retention, moisture content, biodegradability, and the optical properties of the packaging material thereby increasing food shelf life and safety (Farooq et al. 2019; Zhang, Wang, and Cheng 2018). Even though bio‐based edible coatings and food packaging that extend food shelf life exist in the market, there is an urgent need to expand basic research in the field of sustainable and biodegradable edible food packaging. In this context, films based on edible natural polymers offer a valid alternative to materials obtained from oil derivatives (Dmitrenko et al. 2023). Although edible films can never completely replace petroleum derivatives, they have the potential to outperform petroleum‐based plastics as good packaging materials, thus reducing the environmental impact of microplastics (Dmitrenko et al. 2023; Nieto‐Suaza et al. 2019; Sedayu, Cran, and Bigger 2018; Wan Yahaya et al. 2023). Their potential lies both in their intrinsic characteristics and in their capacity to efficiently preserve food through incorporation of antioxidants, antimicrobial agents, enzymes, and functional ingredients (Castro‐García et al. 2022; Dmitrenko et al. 2023). To this end, kappa‐carrageenan isolated from seaweed is a promising hydrocolloid that has a wide range of applications as a gelling agent in cosmetics and the food industry among others. Its excellent film forming property implies that it can be blended with other biopolymers such as starch to overcome its brittleness, low water permeability and resistance in the quest for developing food packaging composites with superior properties (Dmitrenko et al. 2023; Farooq et al. 2022; Tavakoli et al. 2021; Wan Yahaya et al. 2023). However, the reinforcement of carrageenan with starch, which is also a biodegradable polymer, should result in satisfactory properties for forming a packaging material since starch is already a widely accepted biodegradable material for food packaging (Dmitrenko et al. 2023). In the present study, the preparation of carrageenan‐based films incorporating starch was carried out at room temperature after its isolation from *Eucheuma denticulatam*. To better understand the effect of blending the two polymers on the hydrophilic and mechanical properties of the resultant films, the films were then tested for their moisture content, total soluble matter, degree of solubility, water vapor permeability, wettability, mechanical, and optical properties. To evaluate the morphology, structure, transparency, and thermal properties of the obtained films, scanning electron microscopy, Fourier transform infrared spectroscopy, ultraviolet–visible spectroscopy, and thermal gravimetry were utilized, respectively.

## 2 Materials and Methods

### 2.1 Extraction of Refined Carrageenan (RC)

**FIGURE 1:**
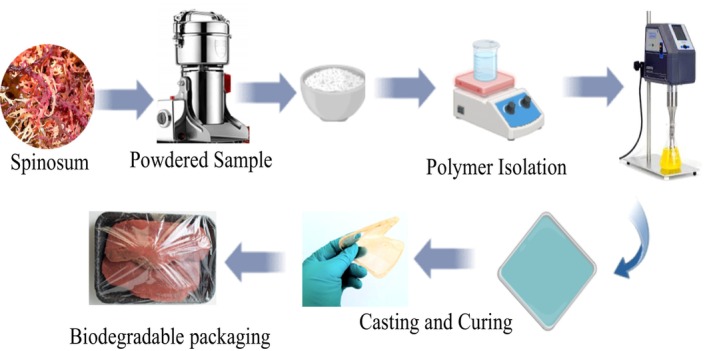
Conversion of starch and seaweed to edible packaging materials.

Dried *Eucheuma denticulatam* seaweed was repeatedly washed with running deionized water to remove sodium chloride and then dried in an oven at 60°C to constant weight (Figure 1). To extract κappa‐carrageenan, two different methods were adopted as described in literature. First, 35 g of dried seaweed in 1 L deionized water was heated at 90°C for 3 h to obtain a thick slurry (Ferreira et al. 2021; Wan Yahaya et al. 2023). The slurry was then homogenized using a household blender to obtain a fine thick viscous solution, which was then added slowly to 95% ethanol to precipitate the polymer, filtered, and dried to constant weight. In the second approach, 35 g of ground seaweed was redispersed in 1 L of 0.01 M KOH solution and heated at 90°C with constant stirring for 3 h. The viscous filtrate was then slowly added to pure ethanol to precipitate the polymer, filtered, and dried to constant weight (Severo et al. 2021).

### 2.2 Preparation of Biocomposite Films

To prepare the composite films, 10 g of dried powdered polymer was dissolved in 200 mL distilled water and heated to a temperature of 80°C with constant stirring until a thick viscous solution was obtained (Castro‐García et al. 2022; Jiang, Cheung, and Ngai 2023; Sedayu, Cran, and Bigger 2018). Then, 1% v/v glycerol was added as a plasticizer and the solution stirred under a constant temperature of 80°C for 15 min, cast in a PTFE mold and dried in an oven to constant weight for film formation. A control sample was prepared in a similar manner but without the addition of glycerol, cast and dried in an oven to constant weight (Ferreira et al. 2021; Wan Yahaya et al. 2023). To prepare carrageenan starch blends, 10 g of powdered carrageenan was dissolved in 250 mL deionized water containing starch (1%–50% w/w), heated to 80°C for 3 h, cast in plastic molds and dried in an oven for 24 h to constant weight (Castro‐García et al. 2022; Jiang, Cheung, and Ngai 2023; Sedayu, Cran, and Bigger 2018).

### 2.3 Hydrophilic Properties

The hydrophilic properties of the films were analyzed by moisture content (MC), dry content (DC), ATRO moisture (AM), swelling degree (SD), total soluble matter (TSM), and water contact angle (CA). MC, DC, AM, SD, and TSM were analyzed using a three‐step gravimetric method; that is, film samples with surface area of 6 cm^2^ were weighed (*M*_1_), dried at 100°C for 24 h and weighed again (*M*_2_) (Janik et al. 2023),

(1) $$\mathrm{MC}=\frac{M_{1}-M_{2}}{M_{1}}\times 100$$

(2) $$\mathrm{DC}=\frac{M_{2}}{M_{1}}\times 100$$

(3) $$\mathrm{AM}=\frac{M_{1}-M_{2}}{M_{2}}\;\times 100$$

To evaluate the degree of solubility (DS) of the films, the samples were then placed in 30 mL of distilled water, left at room temperature for 24 h. This was followed by centrifugation at 3000 rpm to separate the supernatant and the undissolved solid residues, which were then dried and weighed again (M_3_) (Janik et al. 2023; Jiang, Cheung, and Ngai 2023):

(4) $$\mathrm{DS}\left(\%\right)=\frac{M_{3}-M_{2}}{M_{2}}\times 100$$

The total soluble matter of the films was then calculated by difference between the film initial weight and the undissolved residues using Equation (5) (Nieto‐Suaza et al. 2019; Taweechat, Wongsooka, and Rawdkuen 2021):

(5) $$\mathrm{TSM}\;\left(\%\right)=\frac{M_{2}-M_{3}}{M_{2}}\times 100$$

### 2.4 Tensile Strength Measurements of Composite Films

The tensile strength was measured using an in‐house fabricated tensile strength device constructed from a laboratory clamp and stand and metal suspensions (American Society for Testing and Materials 2018; Stevens and Poliks 2003).

**FIGURE 2:**
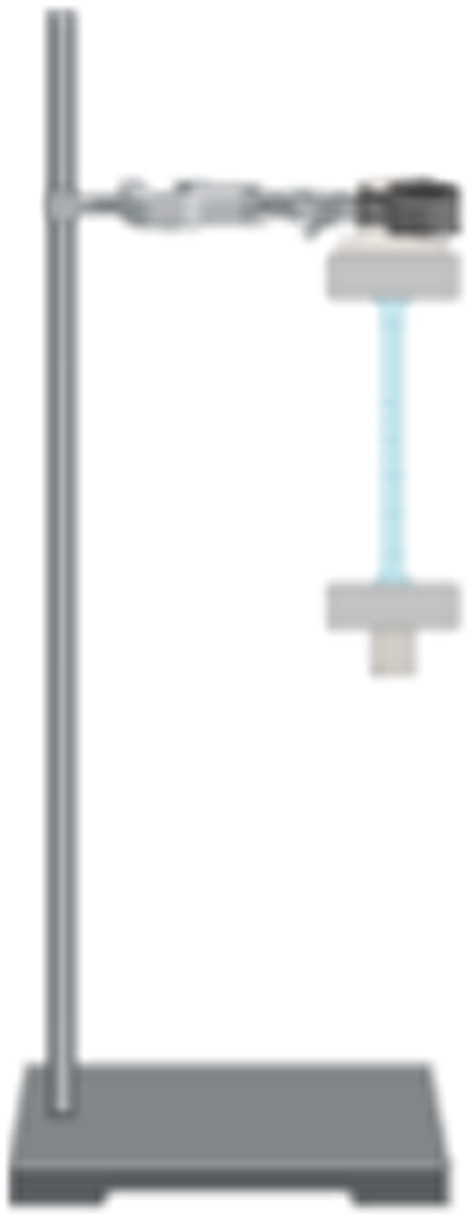
A fabricated tensile strength device adopted from Stevens and Poliks (2003).

The rectangular‐cut (10 mm × 60 mm) film specimens were sandwiched on both ends between two small metal brackets which were then clamped to a slotted mass hanger where successive 5 g increments of weights were added (Figure 2). The extension lengths were recorded after each addition and the total weight at film breakage was recorded. The tensile strength, strain and stress were then calculated using Equations ((6), (7), (8)), respectively (American Society for Testing and Materials 2018; Stevens and Poliks 2003),

(6) $$\mathrm{ts}\;\left(\mathrm{Pa}\right)=\frac{W\left(\mathrm{kg}\right).\left(9.80\frac{N}{\mathrm{kg}}\right)}{A\left(10^{-4}\frac{m^{2}}{{\mathrm{cm}}^{2}}\right)}$$

(7) $$\text{strain}\;\left(\varepsilon \right)=\frac{L-L_{0}}{L_{0}}$$

(8) $$\text{Stress}\left(\sigma \right)\;\left({\mathrm{Nm}}^{-2}\right)=\frac{\text{Load}}{\text{Cross sectional area}}$$

where L = length after applied load, L_0_ = initial length.

### 2.5 Water Vapor Permeability (WVP)

A gravimetric method was used to measure WVP (g m^−1^ s^−1^ Pa^−1^) for the films in triplicate as described in ASTM method E96‐95 with some modifications. Each film sample was sealed over a circular opening 0.003 m^2^ in a flask containing silica gel to obtain a relative humidity of 0% under the flask. This flask was then left in a desiccator containing a saturated solution of sodium chloride to maintain a 75% RH. Water vapor passing through the films and absorbance by the desiccant was then calculated by measuring the increase in weight according to Equation (9) (Dmitrenko et al. 2023; Nieto‐Suaza et al. 2019; Sedayu, Cran, and Bigger 2018; Wan Yahaya et al. 2023):

(9) $$\mathrm{WVP}=\frac{∆m\times l}{A\times t\times P\times ∆\mathrm{RH}}$$

where ∆m is the weight gain of the test cell, l is the film thickness, and A is the exposed area (0.003 m^2^) during duration ∆t under a partial water vapor pressure (P) at (3.169 kPa) and ∆RH is the difference in relative humidity (Dmitrenko et al. 2023; Otenda et al. 2022).

### 2.6 Moisture Absorption (MA)

To determine the films’ moisture absorption, 2cm × 2 cm films were dried at 60°C to constant weight (W_i_) and left in a desiccator with saturated sodium chloride solution to maintain a 75% RH. Samples were weighed after 24 h (W_f_) and the MA was calculated using Equation (10) (Dmitrenko et al. 2023; Nieto‐Suaza et al. 2019; Sedayu, Cran, and Bigger 2018; Wan Yahaya et al. 2023),

(10) $$\mathrm{MA}=\frac{W_{f}-W_{i}}{W_{f}}\times 100$$

### 2.7 Oil Permeability (OP)

To measure the oil film permeability, 4 × 4 cm films were placed onto filter papers dried to constant weight (w_i_) followed by uniform deposition of 25 oil drops (m_oil_) on the film surface for 24 h without leaving the edges of the film. Afterwards, the film with oil was removed, and the filter paper was weighed (w_f_). Oil permeability for the film was then calculated using Equation (11) (Dmitrenko et al. 2023; Nieto‐Suaza et al. 2019; Sedayu, Cran, and Bigger 2018; Wan Yahaya et al. 2023),

(11) $$\mathrm{MA}=\frac{W_{f}-W_{i}}{W_{\text{oil}}}\times 100$$

### 2.8 Opacity Measurement

The opacity of the film was measured using a Shimadzu 1800 UV–Vis spectrophotometer (Shimadzu, Japan) in the wavelength range between 200 and 800 nm. The film was cut into 3 cm × 1 cm and mounted in a cell according to the ASTM D523‐08 (Ferreira et al. 2021; Wan Yahaya et al. 2023). The opacity was then examined at 600 nm and calculated based on Equation (13) (Ferreira et al. 2021; Wan Yahaya et al. 2023),

(13) $$\text{Opacity}=\frac{{\mathrm{Abs}}_{600\;\mathrm{nm}}}{L}$$

where Abs_600_ is the value of absorbance at 600 nm and L is the thickness of film (mm).

### 2.9 Estimation of Trace Metal Concentration Content

The concentration of metal ion present in the seaweed and carrageenan films was evaluated using a Shimadzu 6200 Atomic absorption spectrophotometer (Shimadzu, Japan) after acid digestion. One gram of dried material was digested with 12 mL of HCl: HNO_3_ (3:1) to remove all organic matter from the samples. After digestion, the residue was washed with distilled water, filtered into a 50 mL volumetric flask, and topped to the mark to await analysis (Maina et al. 2019).

### 2.10 Functional Groups Present

The changes in the functional groups present in the films were evaluated by acquiring the IR spectra of the composite using a Bruker Tensor II FT‐IR spectrophotometer model (Bruker, Ettlingen, Germany) in the frequency range of 4000–400 cm^−1^ (Madivoli, Wanakai et al. 2023; Madivoli, Schwarte et al. 2023).

### 2.11 Crystallinity of the Films

Powder X‐ray diffractograms were obtained using a Bruker D8 Advance Diffractometer (Bruker, Ettlingen, Germany) operating a copper tube operating under a voltage and current of 40 kV and 40 mA (Madivoli, Kareru, Gichuki, et al. 2022; Madivoli, Kareru, Gachanja, et al. 2022). The diffractograms were acquired by irradiating the samples with 0.1542 nm monochromatic CuKα radiation between 2θ values of 5°–90° at 0.05° intervals with a measurement time of 1 s per 2θ intervals (Madivoli, Wanakai et al. 2023; Madivoli, Schwarte et al. 2023).

### 2.12 Thermal Profile

The thermal profile of the hydrogels was evaluated using a Mettler Toledo TGA/DSC 30 (Mettler‐Toledo GmbH, Switzerland) (Madivoli, Kareru, Gichuki, et al. 2022; Madivoli, Kareru, Gachanja, et al. 2022). Approximately 5 mg samples were weighed into 40 μL aluminium crucibles, which was then heated from 25°C–500°C and cooled to 25°C.

### 2.13 Surface Morphology and Film Microstructure

Morphological analysis of the hydrogels was observed using Tescan Mira3 LM FE Scanning electron microscope (Tescan, Brno—Kohoutovice, Czech Republic) operating under an accelerating voltage of 3 kV. The samples were sputter coated with 4 nm gold before analysis to avoid charging using AGB 7340 Agar Sputter Coater (Agar Scientific, Essex, United Kingdom) (Kareru, Gichuki, et al. 2022; Madivoli, Kareru, Gachanja, et al. 2022; Madivoli).

## 3 Results and Discussion

### 3.1 Physical Properties

**FIGURE 3:**
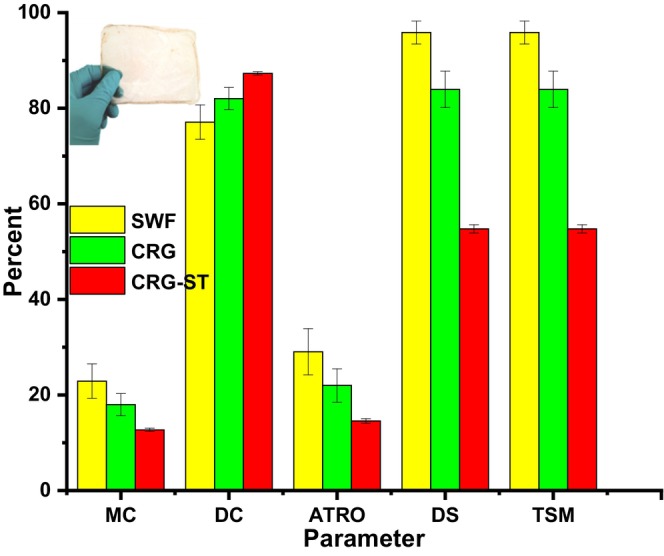
Hydrophilic properties of seaweed (SWF), carrageenan (CRG), and carrageenan‐starch films (CRG‐ST) for food packaging.

Figure 3 depicts the moisture content, ATRO, dry content, total soluble matter (TSM), AND degree of swelling of the composite films.

From Figure 3, it was observed that the MC, ATRO, DS, and TSM of SWF based films was higher as compared to those of CRG and CRG‐ST based films while CRG and CRG‐ST films had a higher DC value. This is as a result of the composition of seaweed which affects their moisture content, degree of swelling, total soluble matter and the ATRO value of the resultant films (Dmitrenko et al. 2023; Nieto‐Suaza et al. 2019; Sedayu, Cran, and Bigger 2018; Wan Yahaya et al. 2023). Interestingly, incorporation of starch in carrageenan matrix resulted in a decrease in the solubility of the CRG‐ST by 50% of its original mass implying that the insoluble fraction was starch. Isolation of carrageenan from seaweed and its subsequent use in preparation of composite films ensure that the resultant films have lower solubility, total soluble matter, and moisture content, which are essential characteristics of a food packaging materials. However, an increase in the dry content ensures that the packing material will be stable for longer periods when exposed to moist conditions without disintegration of the packaging (Dmitrenko et al. 2023; Nieto‐Suaza et al. 2019; Sedayu, Cran, and Bigger 2018; Wan Yahaya et al. 2023). Both CRG and SWF films exhibited DS and TSM values above 80% though CRG had lower DS and TSM values, which implies that both films were very hydrophilic (Figure 3). Never the less, the addition of ST to CRG films reduces the DS and TSM of the resultant CRG‐ST films proportionally to the concentration of ST. The advantage of having highly hydrophilic films lies in the fact that the polymer composite can easily be removed from the environment as they readily dissolve in water. One drawback of high moisture content is that it will limit their application especially when storing food under wet conditions, they encourage microbial growth and limits storage times in moist conditions due to film disintegration (Castro‐García et al. 2022). Similar observations have been reported in other studies. For instance, the addition of ZnO nanopartclies in gelatin films influenced moisture content dynamics, whereas chitosan films containing banana peel extracts had higher moisture content values as compare to neat chitosan films, underlining the impact of biopolymer composition on moisture interaction. In packaging applications, a lower MC value generally indicates reduced hydrophilicity, which can be advantageous in reducing the risk of microbial growth and spoilage.

### 3.2 Mechanical Properties of the Films

**FIGURE 4:**
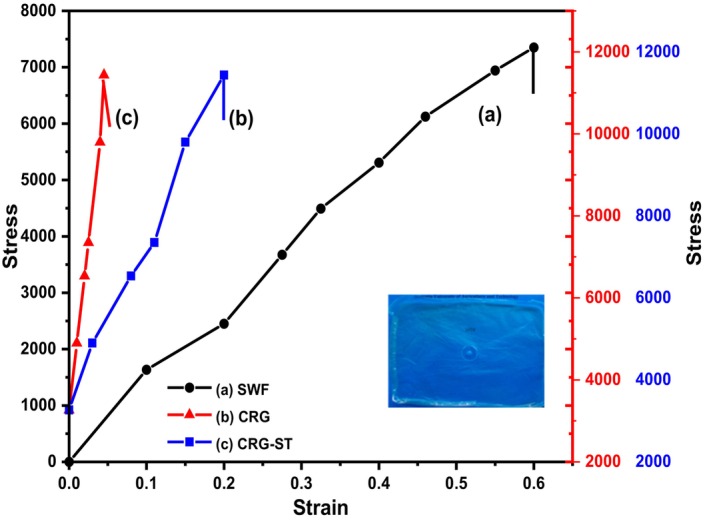
Stress strain curves for (a) SWF (b) CRG (c) CRG‐ST films. Inset CRG‐ST films.

The mechanical properties of the composite films were evaluated in terms of their stress strain curves and the results are depicted in Figure 4.

From the stress–strain curve in Figure 4, it can be observed that carrageenan‐based films were more brittle than seaweed‐based films as the films depicted low percent elongation values during measurement (Singh and Katoch 2020; Taweechat, Wongsooka, and Rawdkuen 2021; Wang et al. 2022). Even though the addition of glycerol as the plasticizer was expected to increase the flexibility and plasticity of CRG films, this was not the case as the percent elongation at break of CRG films was only 4% as compared that of seaweed films with a percent elongation at break of 30%. This decrease in percent elongation can be linked to an increase in hydrogen bond interactions, which have been shown to affect the packing structure of polymer matrix in their solid state as composite films. The low percent elongation values of CRG based films also implies that it had higher strain values as compared to SWF based composite films which was evidenced by its sudden breakage during tensile strength measurements (Figure 4). To overcome this drawback and improve the flexibility of CRG films, incorporation of starch within the matrix resulted in an increase in the strain and the percent elongation of the resultant CRG‐ST films (Figure 4c). de Jesus et al. (2023) reported that carrageenan films incorporating gallic acid had less tensile strength (8.50 ± 0.61 and 10.28 ± 0.65 MPa) and high elongation at break (2.36 ± 0.16 and 1.19% ± 0.17%) compared to the neat samples. Moreover, they also observed that films containing lower concentrations of the polymer exhibited higher stiffness and lower water vapor permeability (de Jesus et al. 2023). It should be noted that the percent elongation at break is a measure of a packaging materials ductility/flexibility and it can be used to evaluate a materials performance under load (Dmitrenko et al. 2023; Nieto‐Suaza et al. 2019; Sedayu, Cran, and Bigger 2018; Wan Yahaya et al. 2023). For instance, low‐density polyethylene films (LDPE), a common food packaging material, exhibits percent elongation values of between 200%–600% while cellulose diacetate pearlescent films exhibit percent elongation values of between 25%–45%. Moreover, polyolefins packaging materials exhibit elongation values of several 100% while rigid plastics exhibit percent elongation values of < 5% (Dmitrenko et al. 2023; Nieto‐Suaza et al. 2019; Sedayu, Cran, and Bigger 2018; Wan Yahaya et al. 2023). Hence, CRG based films are best suited for applications that require rigid polymers such as food containers while SWF films will find applications in areas that require flexible materials such as wrappings and CRG‐ST for packaging materials that have characteristic falling in between the two (Dmitrenko et al. 2023; Nieto‐Suaza et al. 2019; Sedayu, Cran, and Bigger 2018; Wan Yahaya et al. 2023).

### 3.3 Water Vapor Permeability and Moisture Absorption

The water vapor permeability, thickness, moisture absorption, and contact angles of the composite films are depicted in Table 1.

**TABLE 1:** Water vapor permeability (WVP), thickness (l), oil permeability (OP), moisture absorption (MA), and contact angles of the films.

	Thickness (μM)	OP	WVP (gs^−1^m^−1^Pa^−1^)	MA (%)	Contact angle
SWF	100	Nil	2.25 × 10^−7^	29.29 ± 3.5	72.86
CRG	100	Nil	3.65 × 10^−7^	17.29 ± 0.87	80.93
CRG‐ST	100	Nil	2.73 × 10^−7^	23.80 ± 4.12	65.57

**FIGURE 5:**
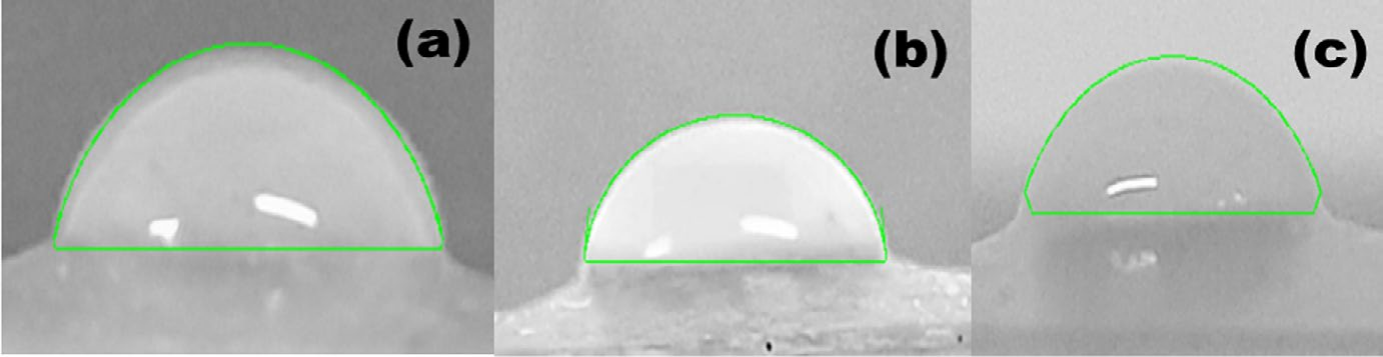
Contact angle measurements for (a) SWF (b) CRG and (c) CRG‐ST.

For a good food packing material, the WVP should be low as possible so as to minimize the transfer of moisture between packed food and the environment. It is an important parameter for active packaging materials because of the role of water in spoilage reactions, keeping food fresh, crisp, or preventing dehydration (Cazón et al. 2022). From Table 1, it can be observed that SWF films had a lower WVP (2.25 × 10^−7^ gs^−1^m^−1^Pa^−1^) compared to CRG (3.65 × 10^−7^ gs^−1^m^−1^Pa^−1^) and a higher moisture absorption of 29.29 ± 3.5 when compared to that of CRG 17.29 ± 0.87 (Marfella et al. 2024; Sedayu, Cran, and Bigger 2018). On the other hand, CRG‐ST films had WVP and MA value that lied between those of CRG and SWF at 2.73 × 10^−7^ gs^−1^m^−1^Pa^−1^ and 23.80 ± 4.12, respectively. Overall, CRG‐ST had lower WVP due to an increase in hydroxyl groups present in the matrix thereby increasing the interaction between water molecules and the films aiding permeation of the water molecules through the film matrix. It has been observed that polysaccharide‐based films have lower WVP permeability as compared to LDPE (6.97 × 10^−12^ ± 7.74 × 10^−13^ gs^−1^m^−1^Pa^−1^) due to their hydrophilic nature (Cazón et al. 2022). Moreover, the lower WVP value can also be attributed to the fact that water molecules were absorbed and entrapped within the film matrix due to the reduced free volume of CRG films. This also explains the higher moisture absorption rate of the SWF films as compared to CRG films as depicted in Table 1. Therefore, the SWG film is suitable as a packaging material for food with low water activity such as dried fruits but not suitable for ones with high moisture such as fish, meat and seafood (Cazón et al. 2022). However, it should also be noted that both films exhibited moisture absorption of less than 30% and no oil permeability hence they can be used in humid environments and for packing oily foods (Marfella et al. 2024; Sedayu, Cran, and Bigger 2018). Wettability implies the determination of contact angles, which determine the wetting degree between a liquid and a solid. Low contact angles values (< 90°) represent good wettability while high contact angles represent poor wettability. In this study, CRG‐ST had the lowest contact angle value, followed by SWF and CRG, which implies that incorporation of ST on CRG films decreased its contact angle values hence improved wettability (Figures 5 and S2, Table 1). The water contact angles of carrageenan‐based films containing nanocellulose *aloe vera* has been shown to be reduced by the addition of starch which resulted in surface hydrophobization (Dmitrenko et al. 2023; Nieto‐Suaza et al. 2019; Sedayu, Cran, and Bigger 2018; Wan Yahaya et al. 2023).

### 3.4 Optical Properties

**FIGURE 6:**
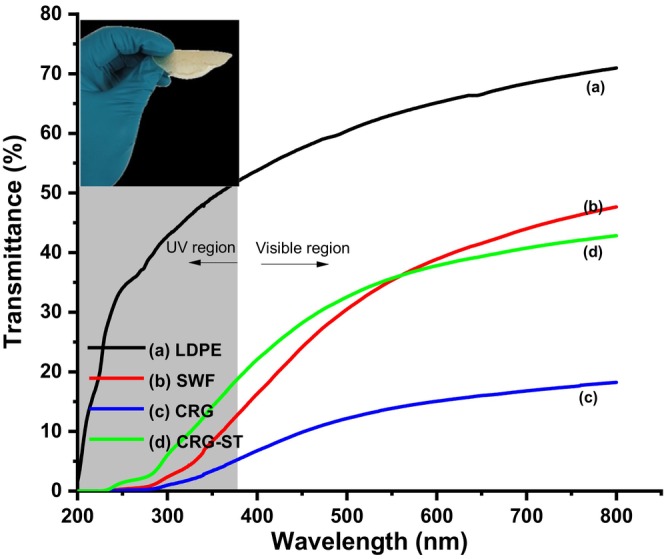
Optical transparency of (a) LDPE, (b) SWF, (c) CRG, and (d) CRG‐ST.

Figure 6 depicts the UV spectra used to evaluate the opacity of the composite films.

As it can be observed from Figure 6, the low‐density polyethylene (LDPE) film displayed high visible light transmittance but poor UV‐shielding properties, while SWF and CRG films were all semi‐transparent but displayed a high UV light shielding property between 200 and 400 nm. Furthermore, CRG films displayed lower visible light transmittance than did SWF films, which might be due to the nature of its rough surface that could cause a scattering of light resulting in lower transparency than SWF films (Jiang, Cheung, and Ngai 2023). Moreover, it is postulated that the strong hydrogen bond interactions between sugar molecules in the polymer chains influences the molecular packing structure in the solid state, hence the observed rigidity, stiffer texture, and less stretchability of CRG films, which leads to a decrease in optical transparency (Sedayu et al. 2021). On the other hand, incorporation of ST within the CRG matrix did not produce a pronounced change in the optical properties of the resultant films in especially in the UV region but it increased the optical transparency in the visible region (Figure 6) (Sedayu et al. 2021). Moreover, the opacity of the films was found to be 18.62, 41.02, 79.89, and 42.23 for LDPE, SWF, CRG, and CRG‐ST, respectively, which implies that CRG films absorbed more radiation and transmitted less. In this case, it also implies that addition of starch, which lowers the opacity of CRG neat films, could be used as a strategy for controlling the opacity of CRG based packaging material (Dmitrenko et al. 2023; Nieto‐Suaza et al. 2019; Sedayu, Cran, and Bigger 2018; Wan Yahaya et al. 2023). Lower opacity values are an important characteristic of food packing as it is linked to food decays and loss in food color and taste, as it reduces the transmission of UV light that leads to a reduction in nutritional loss, oxidative spoilage, and a decrease in food shelf life (Jiang, Cheung, and Ngai 2023; Nieto‐Suaza et al. 2019; Sedayu, Cran, and Bigger 2018; Taweechat, Wongsooka, and Rawdkuen 2021).

### 3.5 Trace Metal Concentrations

**FIGURE 7:**
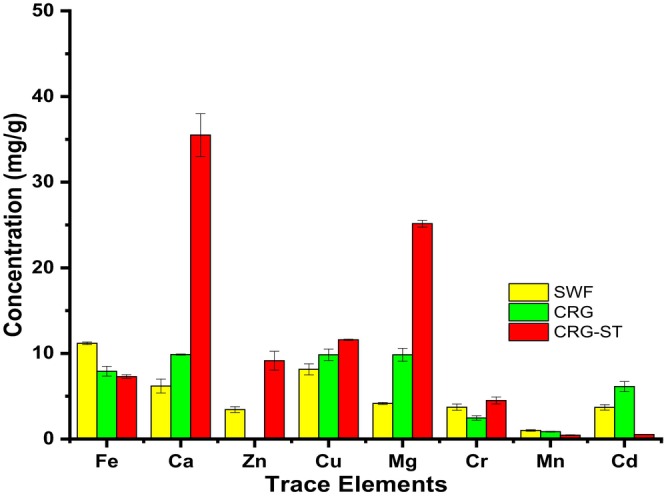
Elemental composition (mg/g) of SWF, CRG, and CRG‐ST films.

Figure 7 depicts the elemental composition for SWF, CRG, and CRG‐ST films.

**FIGURE 8:**
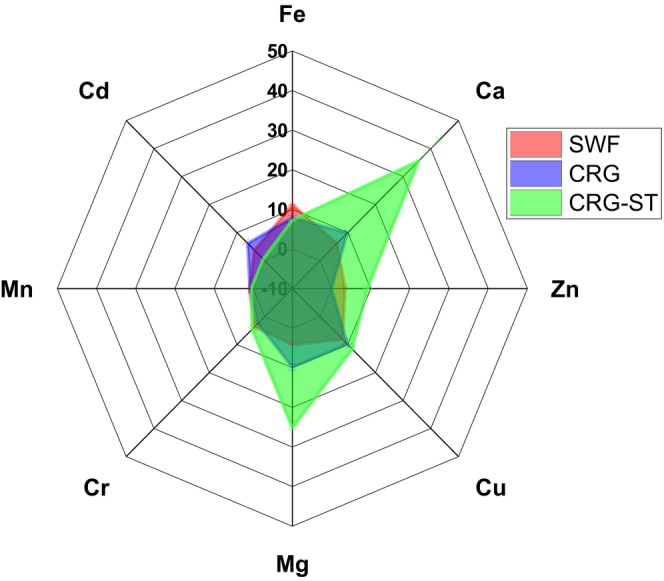
Radar plot depicting elemental composition (mg/g) of SWF, CRG, and CRG‐ST.

From Figures 7 and 8, it can be observed that both SWF films and CRG based films had varying amounts of trace elements, some of which are deemed essential for growth and development. The concentration of iron and chromium were highest in SWF films, while CRG based films had higher concentration of calcium, copper, magnesium, chromium, manganese, and cadmium. These trace elements are required in small amounts for the proper growth, development, and physiology of organisms, and they are used to perform vital metabolic functions in organisms (Cheong et al. 2018; Maina et al. 2019). Hence, it is important to understand which trace metals are present and the levels at which they are present as they determine the edible film's nutritional value thereby influencing the decision to enhance its nutritional content through fortification. Moreover, the concentration of these trace elements was within limits recommended by WHO (Table 2), showing that little risk associated with the consumption of the composite films (Cheong et al. 2018; Maina et al. 2019). According to Berton et al. (2020), commercial carrageenan has a high concentration of metallic ions such as K^+^ = 216.1 g kg^−1^, Na^+^ = 6.3 g kg^−1^, and Ca^2+^ = 12.5 g kg^−1^, which influence the gelling properties of the polymer (Berton et al. 2020; de Jesus et al. 2023).

**TABLE 2:** Recommended dietary trace element allowance for children and adults.

Micronutrient	RDA children mg/day	RDA Male mg/day	RDA Female mg/day
Iron	11.6	8	18
Calcium	1300	1000	1200
Zinc	4.5	12	8
Copper	0.7	0.9	0.9
Magnesium	0–130	400–420	310–320
Chromium	50	0.04	0.03
Manganese	1.9	2.3	1.8
Cadmium	0.2	0.2	0.2

### 3.6 Functional Groups Present

**FIGURE 9:**
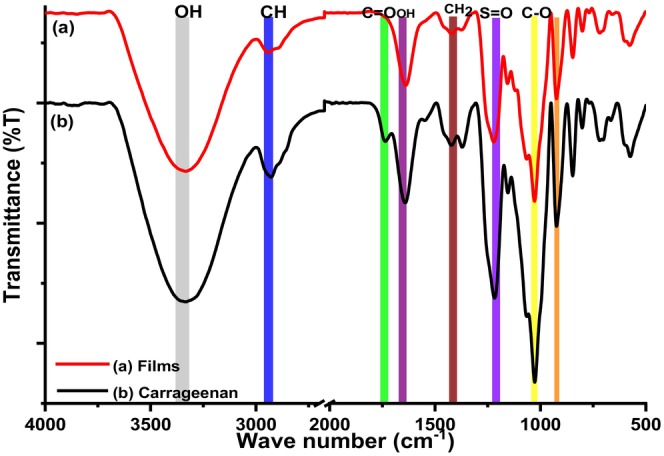
IR spectra of (a) composite films and (b) carrageenan.

**FIGURE 10:**
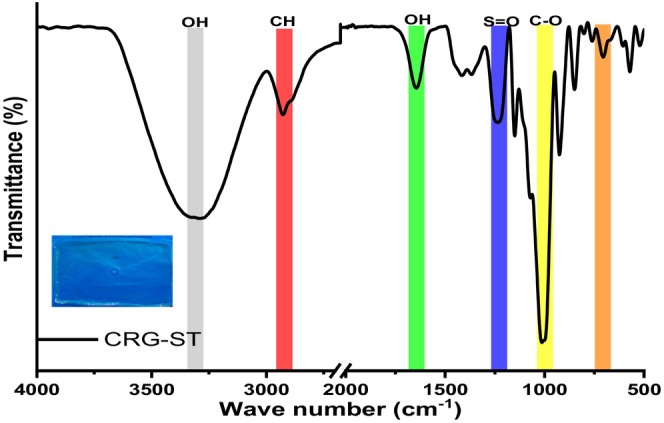
IR spectra of CRG‐ST composite films.

Figures 9 and 10 depicts the changes in functional groups observed during isolation of carrageenan and starch and their subsequent utilization to prepare composite films.

The FTIR spectra of the κ‐carrageenan, carrageenan films, and carrageenan starch films are shown in Figures 9 and 10. For κ‐carrageenan, we observed a –OH broad stretching vibrational band in the region between 3000 and 3600 cm^−1^ (Dmitrenko et al. 2023; Nieto‐Suaza et al. 2019; Sedayu, Cran, and Bigger 2018; Wan Yahaya et al. 2023). The other κ‐carrageenan peaks were observed at 1643 cm^−1^ (polymer bound water), 1241 cm^−1^ (asymmetric stretching of O=S=O), 1069 cm^−1^ (glycosidic bond), 922 cm^−1^ (3,6‐anhydrogalactose group), 847 cm^−1^ (galactose‐4‐sulphate), and 701 cm^−1^ (C_4_—O—S sulphate ester bonding in galactose) (Dmitrenko et al. 2023; Nieto‐Suaza et al. 2019; Sedayu, Cran, and Bigger 2018; Wan Yahaya et al. 2023). It was observed that the spectra of carrageenan and starch (Figure 9) corresponded to those reported in the literature. For all samples, a broad vibrational band between of 3500–3000 cm^−1^ was associated with OH stretching vibration of the hydroxyl group typically present in polysaccharide sugar units, which are responsible for strong hydrogen bond interaction in polysaccharides. Bands in the region 2900–2800 cm^−1^ were attributed to C—H stretching vibrations and are responsible for the material lipophilicity (Figure 10). The vibrational bands in the range between 700 and 1300 cm^−1^ were attributed to the carbohydrate structure and are specific to allow the identification of each polysaccharide (Dmitrenko et al. 2023; Nieto‐Suaza et al. 2019; Sedayu, Cran, and Bigger 2018; Wan Yahaya et al. 2023). Other observed vibrational bands included water absorbed (1600 cm^−1^), carbonyl functional group (around 1750 cm^−1^), sulphate ester vibrational bands, and C—O—C vibrational bands (1020 cm^−1^).

### 3.7 Degree of Crystallinity and Crystal Structure

**FIGURE 11:**
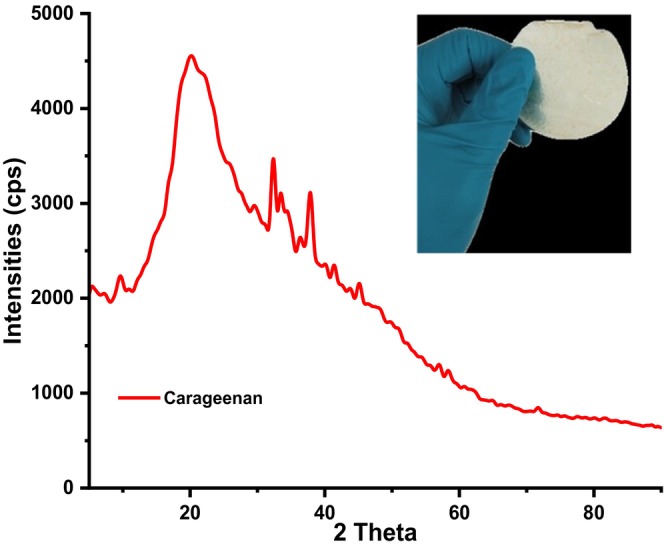
Powder diffractograms of carrageenan composite films.

Figure 11 depicts the powder diffractograms of (a) carrageenan and (b) composite films.

**FIGURE 12:**
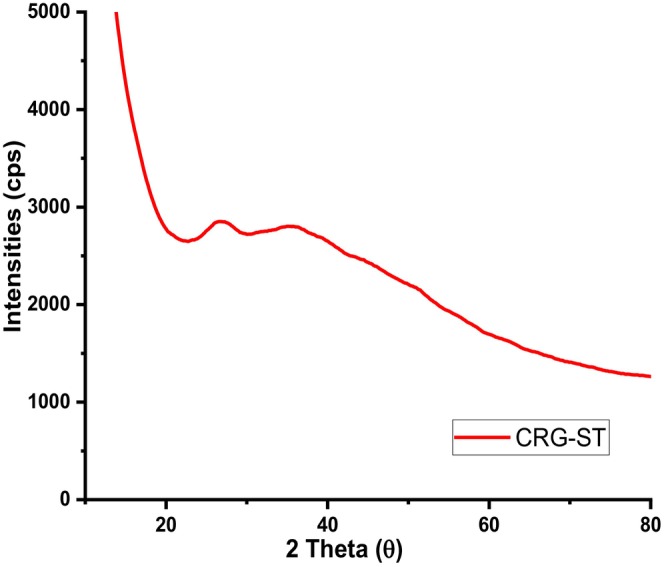
Powder diffractograms of carrageenan—starch films (CRG‐ST).

From Figure 11, powder diffractograms of isolated carrageenan had a broad peak that was centred at 2θ value of 20° and a few sharp peaks centered at 32° and 37°. The presence of the broad peak is associated with the amorphous nature of the polymer while the sharp peaks were associated with residual inorganic impurities embedded in the polymer after its isolation (Castro‐García et al. 2022; Jiang, Cheung, and Ngai 2023; Sedayu, Cran, and Bigger 2018; Sedayu et al. 2021; Simona et al. 2021). The broad hump in the range of 10°–25° reveals its semicrystalline nature. As for CRG‐ST films (Figure 12), the diffractograms revealed a similar pattern as they appeared to be amorphous and lacked sharp peaks observed in neat carrageenan films which are associated with semi‐crystalline materials. Moreover, the intensity of the broad amorphous peak observed in CRG neat films had also reduced this can be linked to the presence of physical interactions in polysaccharides such as hydrogen bonding between the molecular chains and water have a greater impact on their molecular mobility and functional properties. Hence addition of starch in CRG films resulted in changes in the crystallization kinetics, crystalline morphology, crystal forms, and crystallite size of the resultant CRG‐ST films (Figure 12).

### 3.8 Thermal Stability

**FIGURE 13:**
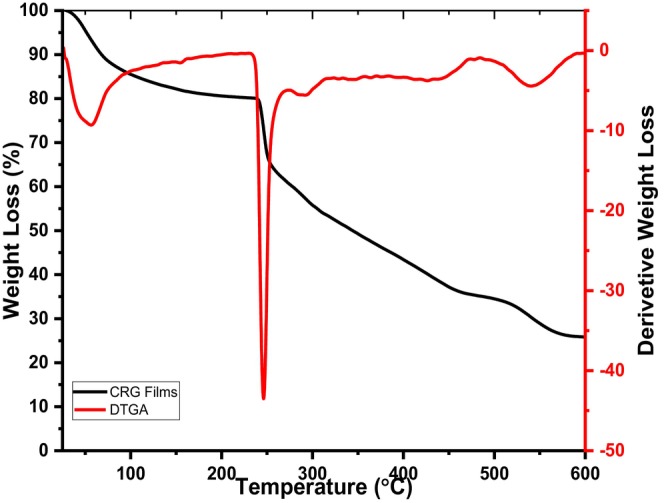
TGA and DTGA thermograms of CRG films.

**FIGURE 14:**
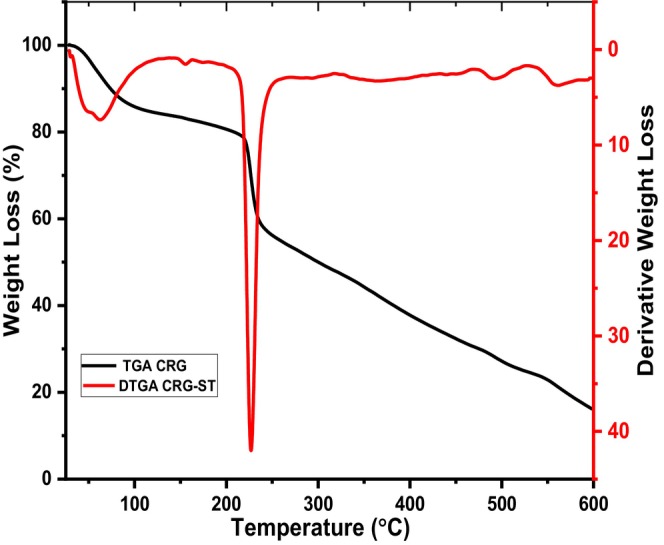
TGA and DTGA thermograms of CRG‐ST films.

Thermal stability of isolated carrageenan was evaluated using TGA, and the results are depicted in Figures 13 and 14.

According to Figures 13 and 14, the TGA and DTGA thermograms of CRG and CRG‐ST films exhibited almost a similar pattern comprising of three degradation cycles at various temperature. The first thermal degradation that was observed below 100°C was associated with decomposition of water and low temperature compounds that are embedded in the film matrix such as volatile compounds (Castro‐García et al. 2022; Jiang, Cheung, and Ngai 2023; Sedayu, Cran, and Bigger 2018; Sedayu et al. 2021; Simona et al. 2021). The second degradation temperature that occurred at 250°C for the case of CRG neat films and 230°C for the case of CRG‐ST films was linked to decomposition of the carbohydrate, where the main weight loss at this temperature corresponds to the high amount of this component (Castro‐García et al. 2022; Jiang, Cheung, and Ngai 2023; Sedayu, Cran, and Bigger 2018; Sedayu et al. 2021; Simona et al. 2021). However, the decrease in the thermal stability of CRG‐ST as compared to CRG was associated with presence of ST molecules in CRG‐ST which resulted in a reduction in molecular interactions between the CRG molecules. Moreover, presence of residual inorganic salts in CRG composite films can also contribute to a higher thermal stability that was observed in CRG composite films (Figure S3). The mass loss at these three degradation cycles were found to be 5%, 73%, 20% for CRG (Figure S3) and 7%, 41%, and 16% for CRG‐ST, respectively. However, TGA thermogram of CRG films was remarkably different from that of carrageenan before films formation, which was characterized by three degradation cycles that occurred at slightly different temperatures and with a higher residual ash content (Figure S3). In this case, the first degradation cycle associated with decomposition of water was observed at 95°C, a second decomposition cycle was observed at 150°C and the last decomposition associated with thermal decomposition of sugar units was observed at 270°C. Interestingly, the TGA thermogram of the composite films were similar to those reported for films developed from other marine algae such as *Gigartina skottsbergii*, *Myriogramme manginii*, *Eucheuma cottonii*, and *Plocamium cartilagineum* (Castro‐García et al. 2022; Jiang, Cheung, and Ngai 2023; Sedayu, Cran, and Bigger 2018; Sedayu et al. 2021; Simona et al. 2021).

### 3.9 Surface Morphology and Microstructure

**FIGURE 15:**
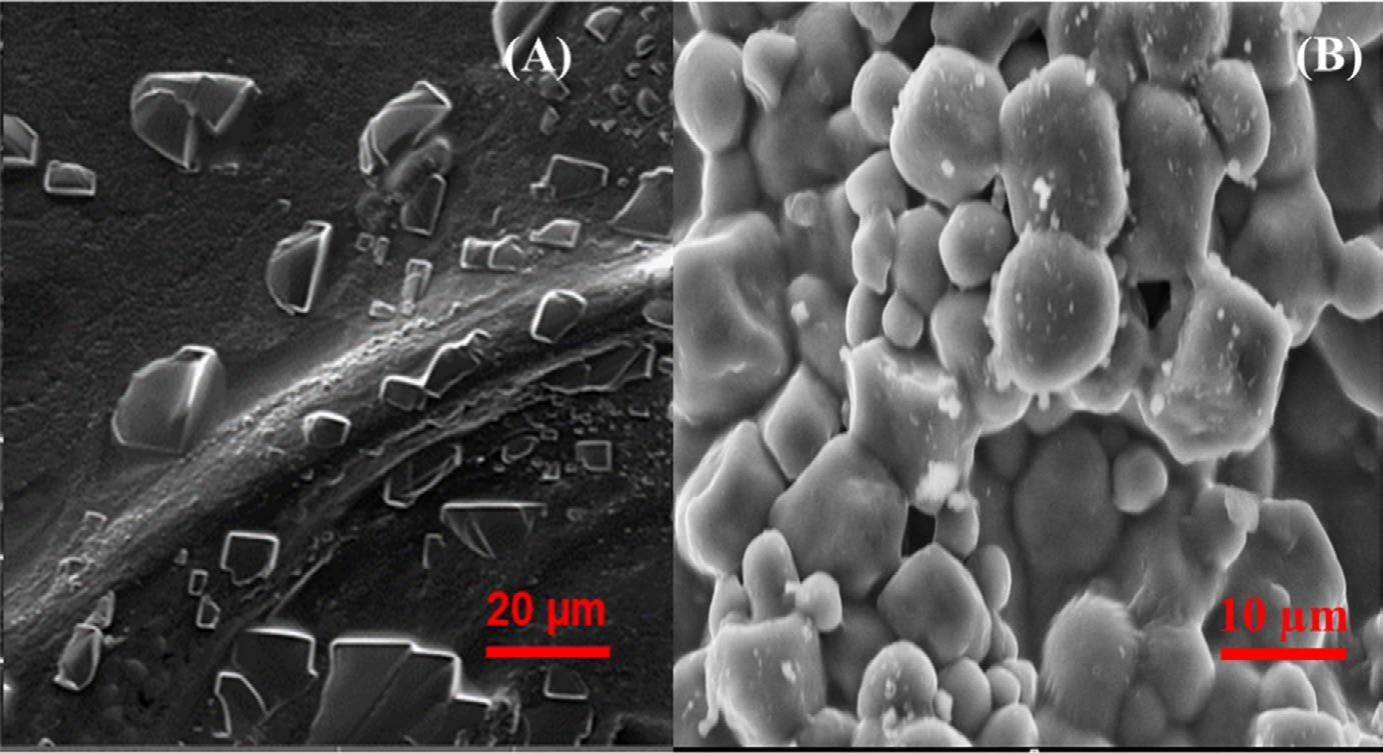
SEM micrographs of CRG neat films and CRG‐ST films.

The surface morphology and microstructure of CRG and CRG‐ST films are depicted in Figure 15.

From Figure 15, it can be observed that CRG neat films comprised sections containing smooth surfaces covered with irregular sheets distributed over the entire film while CRG‐ST films comprised of smooth surface containing what appeared like spherical capsules of different sizes. It has been reported that a film's morphology and microstructure is influenced by the components used in film formulation hence the addition of starch altered the microstructure and morphology of the resultant film whose morphology contained irregular spheres. Higher CRG content has been shown to increase the films surface heterogeneity while at the same time reduces its optical transparency hence the observed morphology (Castro‐García et al. 2022; Jiang, Cheung, and Ngai 2023; Sedayu, Cran, and Bigger 2018; Sedayu et al. 2021; Simona et al. 2021). Furthermore, it has been observed that the extent of surface heterogeneity is inversely proportional to the film transparency, which confirms that heterogeneities (crystals, agglomerates) affect the amount of light passing through or being absorbed by the films, a critical parameter that influences a films ability to absorb UV radiation thereby aiding food preservation (Castro‐García et al. 2022; Jiang, Cheung, and Ngai 2023; Sedayu, Cran, and Bigger 2018; Sedayu et al. 2021; Simona et al. 2021). Moreover, it has been reported that native starch has polyhedral to subspherical shapes granules with a few spots and cracks on the surface of some granules (damaged starch granules). As observed in this study, incorporation of starch in carrageen‐based films influenced the films’ morphology, which appeared to have irregularly shaped spherical particles which can be linked to starch morphology.

## 4 Conclusion

For the prospective development of bio‐based edible food packaging materials, edible and environmentally friendly edible films were developed from carrageenan reinforced with starch. These films developed from carrageenan isolated from *Eucheuma denticulatam* (spinosum) species were highly hydrophilic with remarkable food barrier, mechanical, optical, and thermal properties. They exhibited good water vapor permeability, excellent UV blocking ability, high thermal stabilities, and excellent tensile strength, but poor percent elongation. To enhance their properties, starch powder was added as an additive, which resulted in improved tensile strength, percent elongation, water vapor permeability, and dry content. Moreover, SWF films had a lower WVP compared to CRG and a higher moisture absorption compared CRG. On the other hand, CRG‐ST films had WVP and MA value that lied between those of CRG and SWF respectively. Overall, composite films with different material characteristics can be developed from neat seaweed or carrageenan isolated from seaweed. The film properties can be tuned to meet specific functionalities through the introduction of additives such as starch, which can enhance its properties. Based on these findings, it can be concluded that carrageenan starch composite film might have great potential to be used as a biodegradable edible food packaging material in place of plastic.

## Author Contributions

**E. S. Madivoli:** data curation (equal), formal analysis (equal), investigation (equal), methodology (equal). **J. Kisato:** resources (equal). **P. K. Kimani:** conceptualization (equal), data curation (equal), formal analysis (equal), investigation (equal). **K. Kahinga:** conceptualization (equal), formal analysis (equal), writing – original draft (equal).

## Ethics Statement

The authors have nothing to report.

## Consent

The authors have nothing to report.

## Conflicts of Interest

The authors declare no conflicts of interest.

## Supporting information


Figures S1–S3.

## Data Availability

All data gathered in the scope of the study are embedded within the manuscript.

## References

[fsn34664-bib-0001] American Society for Testing and Materials . 2018. Standard Test Method for Tensile Properties of Thin Plastic Sheeting. West Conshohocken, PA: ASTM International. 10.1520/D0882-18.

[fsn34664-bib-0002] Berton, S. B. R. , G. A. M. de Jesus , R. M. Sabino , et al. 2020. “Properties of a Commercial κ‐Carrageenan Food Ingredient and Its Durable Superabsorbent Hydrogels.” Carbohydrate Research 487: 107883. 10.1016/J.CARRES.2019.107883. 31809910

[fsn34664-bib-0003] Castro‐García, P. G. , S. R. Vasquez‐Garcia , N. Flores‐Ramirez , et al. 2022. “Polymeric Films Prepared From Starch and a Crosslinker Extracted From Avocado Seeds.” Journal of Applied Polymer Science 139, no. 31: 1–11. 10.1002/app.52725.

[fsn34664-bib-0004] Cazón, P. , E. Morales‐Sanchez , G. Velazquez , and M. Vázquez . 2022. “Measurement of the Water Vapor Permeability of Chitosan Films: A Laboratory Experiment on Food Packaging Materials.” Journal of Chemical Education 99, no. 6: 2403–2408. 10.1021/ACS.JCHEMED.2C00449/SUPPL_FILE/ED2C00449_SI_010.DOCX.

[fsn34664-bib-0005] Cheong, K. L. , H. M. Qiu , H. Du , Y. Liu , and B. M. Khan . 2018. “Oligosaccharides Derived From Red Seaweed: Production, Properties, and Potential Health and Cosmetic Applications.” Molecules 23, no. 10: 2451. 10.3390/MOLECULES23102451. 30257445 PMC6222765

[fsn34664-bib-0006] Danopoulos, E. , M. Twiddy , and J. M. Rotchell . 2020. “Microplastic Contamination of Drinking Water: A Systematic Review.” PLoS One 15, no. 7: e0236838. 10.1371/journal.pone.0236838. 32735575 PMC7394398

[fsn34664-bib-0007] de Jesus, G. A. M. , S. B. R. Berton , B. M. Simões , et al. 2023. “κ‐Carrageenan/Poly (Vinyl Alcohol) Functionalized Films With Gallic Acid and Stabilized With Metallic Ions.” International Journal of Biological Macromolecules 253: 127087. 10.1016/J.IJBIOMAC.2023.127087. 37769774

[fsn34664-bib-0008] Dmitrenko, M. , A. Kuzminova , R. M. Cherian , et al. 2023. “Edible Carrageenan Films Reinforced With Starch and Nanocellulose: Development and Characterization.” Sustainability 15, no. 22: 15817. 10.3390/su152215817.

[fsn34664-bib-0009] Eriksen, M. , L. C. M. Lebreton , H. S. Carson , et al. 2014. “Plastic Pollution in the World's Oceans: More Than 5 Trillion Plastic Pieces Weighing Over 250,000 Tons Afloat at Sea.” PLoS One 9, no. 12: 1–15. 10.1371/journal.pone.0111913. PMC4262196 25494041

[fsn34664-bib-0010] Farooq, A. , A. Farooq , S. Jabeen , et al. 2022. “Designing Kappa‐Carrageenan/Guar Gum/Polyvinyl Alcohol‐Based pH‐Responsive Silane‐Crosslinked Hydrogels for Controlled Release of Cephradine.” Journal of Drug Delivery Science and Technology 67: 102969. 10.1016/j.jddst.2021.102969.

[fsn34664-bib-0011] Farooq, M. , T. Zou , G. Riviere , M. H. Sipponen , and M. Österberg . 2019. “Strong, Ductile, and Waterproof Cellulose Nanofibril Composite Films With Colloidal Lignin Particles.” Biomacromolecules 20, no. 2: 693–704. 10.1021/acs.biomac.8b01364. 30358992

[fsn34664-bib-0012] Ferreira, L. F. , L. P. Figueiredo , M. A. Martins , et al. 2021. “Active Coatings of Thermoplastic Starch and Chitosan With Alpha‐Tocopherol/Bentonite for Special Green Coffee Beans.” International Journal of Biological Macromolecules 170: 810–819. 10.1016/J.IJBIOMAC.2020.12.199. 33385457

[fsn34664-bib-0013] Janik, W. , M. Nowotarski , K. Ledniowska , et al. 2023. “Effect of Time on the Properties of Bio‐Nanocomposite Films Based on Chitosan With Bio‐Based Plasticizer Reinforced With Nanofiber Cellulose.” International Journal of Molecular Sciences 24, no. 17: 13205. 10.3390/IJMS241713205. 37686012 PMC10487500

[fsn34664-bib-0014] Jiang, Z. , K. M. Cheung , and T. Ngai . 2023. “Development of Strong and High‐Barrier Food Packaging Films From Cyclic‐Anhydride Modified Bacterial Cellulose.” RSC Sustainability 2, no. 1: 139–152. 10.1039/d3su00219e.

[fsn34664-bib-0015] Kumar, R. , A. K. Vuppaladadiyam , E. Antunes , et al. 2022. “Emerging Contaminants in Biosolids: Presence, Fate and Analytical Techniques.” Emerging Contaminants 8: 162–194. 10.1016/j.emcon.2022.03.004.

[fsn34664-bib-0016] Leslie, H. A. , M. J. M. van Velzen , S. H. Brandsma , A. D. Vethaak , J. J. Garcia‐Vallejo , and M. H. Lamoree . 2022. “Discovery and Quantification of Plastic Particle Pollution in Human Blood.” Environment International 163: 107199. 10.1016/J.ENVINT.2022.107199. 35367073

[fsn34664-bib-0017] Madivoli, E. 2023. “Polysaccharide Based Hydrogels in Drug Delivery Systems, Wound Healing, and Agriculture.” Chemistry Africa 6: 0123456789. 10.1007/s42250-023-00689-1.

[fsn34664-bib-0018] Madivoli, E. , S. I. Wanakai , P. K. Kairigo , and R. S. Odhiambo . 2023. “Encapsulation of AgNPs in a Lignin Isocyanate Film: Characterization and Antimicrobial Properties.” Materials 16, no. 12: 4271. 10.3390/ma16124271. 37374454 PMC10302273

[fsn34664-bib-0019] Madivoli, E. S. , P. G. Kareru , A. N. Gachanja , D. S. Makhanu , and S. M. Mugo . 2022. “Cellulose‐Based Hybrid Nanoarchitectonics With Silver Nanoparticles: Characterization and Antimicrobial Potency.” Journal of Inorganic and Organometallic Polymers and Materials 32, no. 3: 854–863. 10.1007/s10904-021-02212-w.

[fsn34664-bib-0020] Madivoli, E. S. , P. G. Kareru , J. Gichuki , and M. M. Elbagoury . 2022. “Cellulose Nanofibrils and Silver Nanoparticles Enhances the Mechanical and Antimicrobial Properties of Polyvinyl Alcohol Nanocomposite Film.” Scientific Reports 12, no. 1: 1–13. 10.1038/s41598-022-23305-7. 36347953 PMC9643461

[fsn34664-bib-0021] Madivoli, E. S. , J. V. Schwarte , P. G. Kareru , A. N. Gachanja , and K. M. Fromm . 2023. “Stimuli‐Responsive and Antibacterial Cellulose‐Chitosan Hydrogels Containing Polydiacetylene Nanosheets.” Polymers 15, no. 5: 1062. 10.3390/polym15051062. 36904304 PMC10005511

[fsn34664-bib-0022] Maina, E. G. , E. S. Madivoli , J. A. Ouma , J. K. Ogilo , and J. M. Kenya . 2019. “Evaluation of Nutritional Value of Asystasia Mysorensis and Sesamum Angustifolia and Their Potential Contribution to Human Health.” Food Science & Nutrition 7, no. 6: 2176–2185. 10.1002/fsn3.1064. 31289666 PMC6593372

[fsn34664-bib-0023] Marfella, R. , F. Prattichizzo , C. Sardu , et al. 2024. “Microplastics and Nanoplastics in Atheromas and Cardiovascular Events.” New England Journal of Medicine 390, no. 10: 900–910. 38446676 10.1056/NEJMoa2309822 PMC11009876

[fsn34664-bib-0024] Mohanty, A. K. , M. Misra , and L. T. Drzal . 2002. “Sustainable Bio‐Composites From Renewable Resources: Opportunities and Challenges in the Green Materials World.” Journal of Polymers and the Environment 10, no. 1–2: 19–26. 10.1023/A:1021013921916.

[fsn34664-bib-0025] Nieto‐Suaza, L. , L. Acevedo‐Guevara , L. T. Sánchez , M. I. Pinzón , and C. C. Villa . 2019. “Characterization of Aloe Vera‐Banana Starch Composite Films Reinforced With Curcumin‐Loaded Starch Nanoparticles.” Food Structure 22: 100131. 10.1016/J.FOOSTR.2019.100131.

[fsn34664-bib-0026] Okunola, A. A. , I. O. Kehinde , A. Oluwaseun , and E. A. Olufiropo . 2019. “Public and Environmental Health Effects of Plastic Wastes Disposal: A Review.” Journal of Toxicology and Risk Assessment 5, no. 2: 1–13. 10.23937/2572-4061.1510021.

[fsn34664-bib-0027] Otenda, B. V. , P. G. Kareru , E. S. Madivoli , A. M. Salim , J. Gichuki , and S. I. Wanakai . 2022. “Starch‐Hibiscus‐Cellulose Nanofibrils Composite Films as a Model Antimicrobial Food Packaging Material.” Journal of Natural Fibers 19: 1–14. 10.1080/15440478.2022.2058674.

[fsn34664-bib-0028] Rhim, J. W. , H. M. Park , and C. S. Ha . 2013. “Bio‐Nanocomposites for Food Packaging Applications.” Progress in Polymer Science 38, no. 10–11: 1629–1652. 10.1016/j.progpolymsci.2013.05.008.

[fsn34664-bib-0029] Sedayu, B. B. , M. J. Cran , and S. W. Bigger . 2018. “Characterization of Semi‐Refined Carrageenan‐Based Film for Primary Food Packaging Purposes.” Journal of Polymers and the Environment 26, no. 9: 3754–3761. 10.1007/S10924-018-1255-Y/METRICS.

[fsn34664-bib-0030] Sedayu, B. B. , P. Wullandari , A. R. Hakim , and D. Fransiska . 2021. “Initial Properties Identification of Refined‐and Semi Refined‐Carrageenans as Raw Materials for Biodegradable Plastic Production.” Squalen Bulletin of Marine and Fisheries Postharvest and Biotechnology 16, no. 2: 57–64. 10.15578/squalen.533.

[fsn34664-bib-0031] Severo, C. , I. Anjos , V. G. L. Souza , et al. 2021. “Development of Cranberry Extract Films for the Enhancement of Food Packaging Antimicrobial Properties.” Food Packaging and Shelf Life 28: 100646. 10.1016/j.fpsl.2021.100646.

[fsn34664-bib-0032] Shankar, S. , L. F. Wang , and J. W. Rhim . 2016. “Preparations and Characterization of Alginate/Silver Composite Films: Effect of Types of Silver Particles.” Carbohydrate Polymers 146: 208–216. 10.1016/j.carbpol.2016.03.026. 27112867

[fsn34664-bib-0033] Simona, J. , D. Dani , S. Petr , N. Marcela , T. Jakub , and T. Bohuslava . 2021. “Edible Films From Carrageenan/Orange Essential Oil/Trehalose—Structure, Optical Properties, and Antimicrobial Activity.” Polymers 13, no. 3: 1–19. 10.3390/polym13030332. PMC7864528 33494246

[fsn34664-bib-0034] Singh, G. , and M. Katoch . 2020. “Antimicrobial Activities and Mechanism of Action of Cymbopogon Khasianus (Munro Ex Hackel) Bor Essential Oil.” BMC Complementary Medicine and Therapies 20, no. 1: 1–9. 10.1186/s12906-020-03112-1. 33153473 PMC7643435

[fsn34664-bib-0035] Stevens, E. S. , and M. D. Poliks . 2003. “Tensile Strength Measurements on Biopolymer Films.” Journal of Chemical Education 80, no. 7: 810–812. 10.1021/ed080p810.

[fsn34664-bib-0036] Tavakoli, S. , M. Kharaziha , S. Nemati , and A. Kalateh . 2021. “Nanocomposite Hydrogel Based on Carrageenan‐Coated Starch/Cellulose Nanofibers as a Hemorrhage Control Material.” Carbohydrate Polymers 251: 117013. 10.1016/j.carbpol.2020.117013. 33142576

[fsn34664-bib-0037] Taweechat, C. , T. Wongsooka , and S. Rawdkuen . 2021. “Properties of Banana (Cavendish spp.) Starch Film Incorporated With Banana Peel Extract and Its Application.” Molecules 26, no. 5: 1406. 10.3390/MOLECULES26051406. 33807750 PMC7961874

[fsn34664-bib-0038] Visakh, P. M. , A. P. Mathew , and S. Thomas . 2013. “Natural Polymers: Their Blends, Composites and Nanocomposites: State of Art, New Challenges and Opportunities.” In Advanced Structured Materials, vol. 18, 1–20. Berlin, Heidelberg: Springer. 10.1007/978-3-642-20940-6_1.

[fsn34664-bib-0039] Wagner, M. , L. Monclús , H. P. H. Arp , et al. 2024. “State of the Science on Plastic Chemicals–Identifying and Addressing Chemicals and Polymers of Concern.”

[fsn34664-bib-0040] Wan Yahaya, W. A. , N. A. M. Azman , F. Adam , S. D. Subramaniam , K. H. Abd Hamid , and M. P. Almajano . 2023. “Exploring the Potential of Seaweed Derivatives for the Development of Biodegradable Plastics: A Comparative Study.” Polymers 15, no. 13: 2884. 10.3390/POLYM15132884. 37447534 PMC10346765

[fsn34664-bib-0041] Wang, W. , Y. Gong , Q. Sun , L. Li , A. Xu , and R. Liu . 2022. “High Performance Polyvinyl Alcohol/Polylactic Acid Materials: Facile Preparation and Improved Properties.” Journal of Applied Polymer Science 139, no. 26: e52470. 10.1002/app.52470.

[fsn34664-bib-0042] Worm, B. , H. K. Lotze , I. Jubinville , C. Wilcox , and J. Jambeck . 2017. “Plastic as a Persistent Marine Pollutant.” Annual Review of Environment and Resources 42: 1–26. 10.1146/annurev-environ-102016-060700.

[fsn34664-bib-0043] Wu, Z. , W. Deng , J. Luo , and D. Deng . 2019. “Multifunctional Nano‐Cellulose Composite Films With Grape Seed Extracts and Immobilized Silver Nanoparticles.” Carbohydrate Polymers 205: 447–455. 10.1016/j.carbpol.2018.10.060. 30446127

[fsn34664-bib-0044] Yang, H. , G. Chen , and J. Wang . 2021. “Microplastics in the Marine Environment: Sources, Fates, Impacts and Microbial Degradation.” Toxics 9, no. 2: 1–19. 10.3390/toxics9020041. PMC7927104 33671786

[fsn34664-bib-0045] Zhang, R. , X. Wang , and M. Cheng . 2018. “Preparation and Characterization of Potato Starch Film With Various Size of Nano‐SiO2.” Polymers 10, no. 10: 9–12. 10.3390/POLYM10101172. PMC6403978 30961097

