# Supplementary material for: Evaluation of the Food Barrier and Mechanical Properties of Carrageenan‐Starch Composite Films
Source: Food Sci Nutr. 2024 Dec 9;13(1):e4664. doi: 10.1002/fsn3.4664 (PMC11717021; doi:10.1002/fsn3.4664)
Supplement: Supplementary file 1 — Figures S1–S3. [file FSN3-13-e4664-s001.docx]

**Supplementary Materials**

**Figure S1:** IR spectra of composite films prepared using carrageenan isolated using (a) 0.1 M KOH (b) deionized water

**
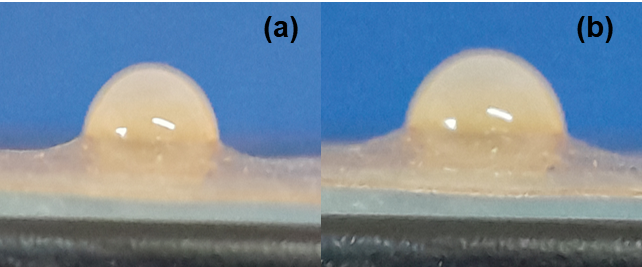
**

**Figure S2:** Contact angle measurements of (a) CRG and (b) CRG-ST

**Figure S3:** TGA and DTGA thermograms of carrageenan isolated from red seaweed
